# The Association of Ascorbic Acid, Deferoxamine and N-Acetylcysteine Improves Cardiac Fibroblast Viability and Cellular Function Associated with Tissue Repair Damaged by Simulated Ischemia/Reperfusion

**DOI:** 10.3390/antiox8120614

**Published:** 2019-12-03

**Authors:** Pablo Parra-Flores, Jaime A Riquelme, Paula Valenzuela-Bustamante, Sebastian Leiva-Navarrete, Raúl Vivar, Jossete Cayupi-Vivanco, Esteban Castro, Claudio Espinoza-Pérez, Felipe Ruz-Cortés, Zully Pedrozo, Sergio Lavandero, Ramon Rodrigo, Guillermo Diaz-Araya

**Affiliations:** 1 Laboratorio de Farmacología Molecular, Departamento de Química Farmacológica y Toxicológica, Facultad de Ciencias Químicas y Farmacéuticas, Universidad de Chile, Santos Dumont 964, Independencia, Santiago 8380492, Chile; qfpablo@gmail.com (P.P.-F.); p.valenzuelabustamante@gmail.com (P.V.-B.); jossete.c.v@gmail.com (J.C.-V.); ecastro.chl@gmail.com (E.C.); claudio.espinoza06@gmail.com (C.E.-P.); felipe.ruz.cortes@gmail.com (F.R.-C.); 2 Advanced Center for Chronic Diseases (ACCDiS), Facultad de Ciencias Químicas y Farmacéuticas and Facultad de Medicina, Universidad de Chile, Santos Dumont 964, Independencia, Santiago 8380492, Chile; riquelme@ciq.uchile.cl (J.A.R.); srleiva.navarrete@gmail.com (S.L.-N.); zpedrozo@med.uchile.cl (Z.P.); slavander@uchile.cl (S.L.); 3 Instituto de Ciencias Biomédicas, Facultad de Medicina, Universidad de Chile, Independencia 1027, Independencia, Santiago 8380453, Chile; raulvivar@med.uchile.cl (R.V.); rrodrigo@med.uchile.cl (R.R.); 4 Cardiology Division, Department of Internal Medicine, University of Texas Southwestern Medical Center, Dallas, TX 75390-8573, USA

**Keywords:** ascorbic acid, deferoxamine, N-acetylcysteine, ischemia/reperfusion, cardiac fibroblasts, reactive oxygen species

## Abstract

Acute myocardial infarction is one of the leading causes of death worldwide and thus, an extensively studied disease. Nonetheless, the effects of ischemia/reperfusion injury elicited by oxidative stress on cardiac fibroblast function associated with tissue repair are not completely understood. Ascorbic acid, deferoxamine, and N-acetylcysteine (A/D/N) are antioxidants with known cardioprotective effects, but the potential beneficial effects of combining these antioxidants in the tissue repair properties of cardiac fibroblasts remain unknown. Thus, the aim of this study was to evaluate whether the pharmacological association of these antioxidants, at low concentrations, could confer protection to cardiac fibroblasts against simulated ischemia/reperfusion injury. To test this, neonatal rat cardiac fibroblasts were subjected to simulated ischemia/reperfusion in the presence or absence of A/D/N treatment added at the beginning of simulated reperfusion. Cell viability was assessed using trypan blue staining, and intracellular reactive oxygen species (ROS) production was assessed using a 2′,7′-dichlorofluorescin diacetate probe. Cell death was measured by flow cytometry using propidium iodide. Cell signaling mechanisms, differentiation into myofibroblasts and pro-collagen I production were determined by Western blot, whereas migration was evaluated using the wound healing assay. Our results show that A/D/N association using a low concentration of each antioxidant increased cardiac fibroblast viability, but that their separate administration did not provide protection. In addition, A/D/N association attenuated oxidative stress triggered by simulated ischemia/reperfusion, induced phosphorylation of pro-survival extracellular-signal-regulated kinases 1/2 (ERK1/2) and PKB (protein kinase B)/Akt, and decreased phosphorylation of the pro-apoptotic proteins p38- mitogen-activated protein kinase (p38-MAPK) and c-Jun-N-terminal kinase (JNK). Moreover, treatment with A/D/N also reduced reperfusion-induced apoptosis, evidenced by a decrease in the sub-G1 population, lower fragmentation of pro-caspases 9 and 3, as well as increased B-cell lymphoma-extra large protein (Bcl-xL)/Bcl-2-associated X protein (Bax) ratio. Furthermore, simulated ischemia/reperfusion abolished serum-induced migration, TGF-β1 (transforming growth factor beta 1)-mediated cardiac fibroblast-to-cardiac myofibroblast differentiation, and angiotensin II-induced pro-collagen I synthesis, but these effects were prevented by treatment with A/D/N. In conclusion, this is the first study where a pharmacological combination of A/D/N, at low concentrations, protected cardiac fibroblast viability and function after simulated ischemia/reperfusion, and thereby represents a novel therapeutic approach for cardioprotection.

## 1. Introduction

Ischemic heart disease is still one of the leading causes of mortality in the world [1]. Prolonged myocardial ischemia due to partial or complete coronary artery occlusion generates an imbalance between the supply and demand of O_2_, which subsequently leads to cardiac cell death and thus, to development of acute myocardial infarction (MI) [2,3]. Restoration of blood flow is of utmost importance for myocardial salvage. Nevertheless, this procedure itself induces necrotic and apoptotic cell death. This paradoxical effect is known as lethal reperfusion injury and can contribute up to 50% of final infarct size [4,5]. One of the key mediators of reperfusion injury is oxidative stress, characterized by a massive release of reactive oxygen species (ROS) at the beginning of reperfusion, which triggers cellular lipid peroxidation, as well as protein/nucleic acid oxidation, and consequently, activates pro-apoptotic pathways associated with p38-MAPK and JNK proteins [6,7]. In addition, ROS of enzymatic origin can be produced by xanthine oxidase, reduced nicotinamide adenine dinucleotide phosphate hydrogen (NADPH) oxidase, and uncoupled endothelial nitric oxide synthase (eNOS), whereas non-enzymatic generation of ROS may arise from uncoupled mitochondrial electron transport chain and Fenton and Haber-Weiss reactions mediated by free iron from cell lysis [6,8].

After myocardial damage caused by ischemia/reperfusion (I/R), the tissue repair processes are mainly coordinated by cardiac fibroblasts (CF) [9], which are the second most numerous non-myocyte population in the mouse heart [10]. Under physiological conditions, these cells can synthesize and degrade collagen to provide a constant replacement of the myocardial extracellular matrix (ECM). Moreover, CF secrete a variety of paracrine factors that may regulate cellular function in cardiomyocytes, the endothelium, vascular smooth muscle cells, and immune system cells [11]. In addition, upon pathophysiological stimuli, CF migrate, proliferate, secrete pro-inflammatory mediators, metalloproteases, and differentiate into cardiac myofibroblasts (CMF) to promote scar tissue formation [9].

Although previous studies have evaluated the effect of simulated I/R (sI/R) on CF death [12,13,14,15], the impact of oxidative stress triggered by reperfusion injury on the functional capacity associated with fibroblast-mediated tissue repair, remains to be fully elucidated. In this context, the use of antioxidants to confer cardioprotection in patients with MI subjected to percutaneous coronary angioplasty has been previously reviewed [6,8,16]. Ascorbic acid (A), deferoxamine (D), and N-acetylcysteine (N) are antioxidants that can separately act as a free radical scavenger, a free iron chelator and a reduced glutathione (GSH) precursor, respectively. In ex vivo and in vivo models of MI these three antioxidants can protect against myocardial reperfusion injury and prevent the death of cardiomyocytes exposed to sI/R [17,18,19,20,21,22]. However, the effects of these antioxidants, either separate or combined, on the viability of CF after sI/R and the potential mechanisms associated with this protection have not been thoroughly explored.

The aim of this study was to determine whether the pharmacological association of A/D/N, at low concentrations, protects CF from death and recovers cellular function associated with tissue repair processes after damage by sI/R; along with elucidating the signaling pathways that mediate this effect.

## 2. Material and Methods

### 2.1. Reagents

Dulbecco’s Modified Eagle’s medium F12 (DMEM-F12; No 12500062), alamarBlue™ Cell Viability Reagent (Resazurin; No DAL1100), and Collagenase, Type II, powder (No 17101015) were obtained from Thermo Fisher Scientific (Waltham, MA, USA). Fetal Bovine Serum (FBS; No 04-001-1A), Trypsin ethylenediaminetetraacetic acid (EDTA; 0.5%), EDTA 0.2% (10X Solution (No 03-051-5B), penicillin-streptomycin-amphotericin B Solution (No 03-033-1B), and Trypan Blue (0.5%) Solution (No 03-102-1B) were obtained from Biological Industries (Cromwell, CT, USA). Recombinant human TGF-beta 1 protein (TGF-β1; No 240-B) was obtained from R & D Systems, Inc. (Minneapolis, MN, USA). Pascorbin (vitamin C infusion bottle) was obtained from Pascoe Naturmedizin (Giessen, Germany). M199 medium (No M2520), pancreatin from porcine pancreas (No P3292), RNAse A (No 10109142001), propidium iodide (No P4170), Bradford reagent (No B6916), RIPA lysis and extraction buffer (No 89900), Halt™ Protease Inhibitor Cocktail (100X, No 78438), Halt™ Phosphatase Inhibitor Cocktail (No 78427), enhanced chemiluminescence (ECL) western blotting detection reagents (No GERPN2209), deferoxamine mesylate salt (No D9533), N-Acetyl-L-cysteine (No A7250), 2′,7′-Dichlorofluorescin diacetate (DCFH-DA; No D6883), 5-Bromo-2′-deoxyuridine (BrdU; No B5002), angiotensin II human (No A9525), and crystal violet (No C0775) were obtained from Sigma-Aldrich Corp. (St. Louis, MI, USA). All inorganic salt products and methanol (No 106035) were obtained from Merck (Darmstadt, Germany). Prestained Protein Ladder-Broad molecular weight (10–245 kDa; No ab116028) was obtained from Abcam (Cambridge, MA, USA). Nitrogen gas (N_2_) cylinders were obtained from Linde Group (Santiago, Chile). Primary antibodies for phospho-ERK 1/2 (p-ERK1/2, No 9101), ERK1/2 (No 9102), phospho-Akt (p-Akt, No 9271), Akt (No. 9272), phospho-p38-MAPK (p-p38-MAPK, No 9211), p38 (No 9212), phospho-JNK (p-JNK, No 9251), JNK (No 9252), Pro-caspase 3 (No 9662), Pro-caspase 9 (No 9508), Bax (No 2772), Bcl-xl (No 2764), alpha-smooth muscle actin (α-SMA; No 19245), alpha-1 type 1 collagen (COL1A1; No 84336), glyceraldehyde 3-phosphate dehydrogenase (GAPDH; No 5174), and secondary antibodies for anti-rabbit IgG (No 7074) and anti-mouse IgG (No 7076) conjugated with horseradish peroxidase (HRP) were obtained from Cell Signaling Technology (Danvers, MA, USA). All plastic material was obtained from Corning Incorporated (Corning, NY, USA).

### 2.2. Animals

Sprague-Dawley neonatal rats (1 to 3-days-old) were obtained from Animal Breeding Facilities of the Facultad de Ciencias Químicas y Farmacéuticas (Universidad de Chile, Santiago, Chile). All studies followed the Guide for the Care and Use of Laboratory Animals (8th edition, 2011) [23], and International Guiding Principles for Biomedical Research Involving Animals, as issued by the Council for the International Organizations of Medical Sciences [24]. Experimental protocols were approved by the Bioethics Committee for Animal Research from the Facultad de Ciencias Químicas y Farmacéuticas, Universidad de Chile (CBE2017-08).

### 2.3. Isolation and Culture of Cardiac Fibroblasts

Neonatal rat CF were isolated as previously described [12]. In a sterile zone, rats were swiftly decapitated and their hearts rapidly excised. After removing the atrium, ventricles were minced and digested with collagenase (0.05% *w/v*) and pancreatin (0.05% *w/v*). Cells were pre-cultured on plastic dishes of 100 mm diameter, with DMEM-F12/M199 (4:1) medium containing FBS (10%) and penicillin-streptomycin-amphotericin B and kept in an incubator with O_2_ (95%) and CO_2_ (5%) at 37 °C. CF differentially adhered on plastic dishes, which separated them from cardiomyocytes. After 3 h, CF were washed three times with sterile phosphate buffer saline (PBS) and culture medium was replenished. Next, CF were incubated during 3–5 days, until confluence was reached. Afterwards, medium was replaced by DMEM-F12 containing FBS (3%) and penicillin-streptomycin-amphotericin B. Cells underwent up to a maximum of two passages and detachment was performed using trypsin EDTA (0.5%), EDTA 0.2% (1X), followed by protease inhibition with DMEM-F12 containing FBS (10%). CF were then collected and seeded on plastic plates in DMEM-F12 medium without FBS for 24 h before experiments.

### 2.4. Isolation and Culture of Cardiomyocytes

Isolation of neonatal rat ventricular cardiomyocytes was performed using one- to three-day-old Sprague-Dawley rats, as described previously [25]. After enzymatic digestion of the myocardium with collagenase 0.02% and pancreatin 0.06%, cells were pre-plated to discard non-myocyte cells and the myocyte-enriched fraction was plated on gelatin-precoated 35 mm dishes and grown in DMEM/M199 (4:1) medium with 10% (*w/v*) fetal bovine serum (FBS) and 100 mM bromodeoxyuridine for 24 h before the experiments.

### 2.5. Simulated Ischemia/Reperfusion (sI/R) and Antioxidant Treatment

We used a modified protocol from Vivar et al. [13]. Following 24 h serum deprivation, cells were washed two times with PBS before sI/R. Cell medium was replaced with ischemic buffer, which contained: NaCl (115 mM), KCl (12 mM), MgCl_2_ (1.2 mM), CaCl_2_ (2 mM), (4-(2-hydroxyethyl)-1-piperazineethanesulfonic acid (HEPES) (25 mM), and lactic acid (20 mM), pH 6.2. For simulated ischemia, hypoxia was achieved by placing the cells in a customized chamber (STEMCELL Technologies Inc., Vancouver, Canada) with < 1% O_2_ and 99% N_2_ environment at 37 °C for 6 h. Simulated reperfusion was performed replacing the ischemic medium with DMEM-F12 medium and placing cells with O_2_ (95%) and CO_2_ (5%) at 37 °C for 16 h. At the onset of simulated reperfusion CF, were treated separately with A, D, and N using 10,000, 1000, 100, 10, and 1 µM each for preliminary cell viability studies. Antioxidants were then, administered in different combinations at 10 or 1 µM each. In later experiments, the A/D/N association was added using 10 μM of each antioxidant and simulated reperfusion time was variable, depending on the experiment. Control cells were incubated under normoxic conditions in DMEM-F12 medium and kept in an incubator with O_2_ (95%) and CO_2_ (5%) at 37 °C for the exact duration of simulated ischemia and sI/R experiments.

### 2.6. Cell Viability with Trypan Blue

CF at a 156 cells/mm^2^ density on 35 mm plastic dishes were used to assess viability using trypan blue (0.5%) staining. After 16 h simulated reperfusion, cells were washed two times with PBS and treated with trypsin EDTA (0.5%), EDTA 0.2% (1×) to detach cells, followed by administration of FBS (10%) to induce inactivation. After collecting the cells, aliquots of 20 μL of sample, plus 20 μL of trypan blue (0.5%) reagent were homogenized, and then 8 μL were transferred to a Neubauer chamber (Paul Marienfeld GmbH & Co. KG, Lauda-Königshofen, Germany) to count viable cells (unstained) using optic microscopy. Cell viability was quantified as a percentage (%) of number of cells after 6 h normoxia (100%). A minimum of 1000 cells/mL was counted in each sample.

### 2.7. Cell Viability with the Resazurin Reduction Assay

Cells were seeded in clear bottom 24-well plates with a density of 263 cells/mm^2^. After 16 h simulated reperfusion, culture medium was replaced by DMEM-F12, followed by incubation with resazurin (10%) for 4 h. Viable cells with active metabolism can reduce the non-fluorescent resazurin to fluorescent resorufin. Fluorescence was measured at 585 nm (λ excitation) and 570 nm (λ emission) in a BioTek™ Synergy™ 2 Multi-Mode Microplate Reader (BioTek Instruments, Inc., Winooski, VT, USA).

### 2.8. Determination of Intracellular ROS

Cells were seeded in clear bottom 24-well plates with a density of 421 cells/mm^2^. DCFH-DA is a non-fluorescent cell membrane permeable probe which is de-esterified intracellularly and then oxidized to the fluorescent 2′,7′-dichlorofluorescein. At the end of 6 h simulated ischemia, cells were incubated with DCFH-DA (20 µM) dissolved in fresh ischemic medium for 45 min at 37 °C. Rapidly, cells were washed two times with PBS, and then DMEM-F12, with or without antioxidants, was added. Fluorescence intensity was measured during the first 30 min of simulated reperfusion at 485 nm (λ excitation) and 535 nm (λ emission) in a BioTek™ Synergy™ 2 Multi-Mode Microplate Reader (BioTek Instruments, Inc., Winooski, VT, USA).

### 2.9. Necrosis Assessment by Flow Cytometer

CF were seeded with a 106 cells/mm^2^ density on 60 mm plastic dishes. After 16 h of simulated reperfusion, dead cells were collected from medium, centrifuged at 252× *g* for 5 min, and kept at 4 °C. Live cells were detached from plates using trypsin EDTA (0.5%), EDTA 0.2% (1X), and mixed with the pellet of dead cells. Subsequently, propidium iodide (1 mg/mL) was added and necrotic cell death was evaluated by flow cytometry in a BD FACSCantoA (Becton Dickinson & Company, Franklin Lakes, NJ, USA). A total of 5000 cells/sample were analyzed.

### 2.10. Sub-G1 Population Determination by Flow Cytometry

Cells were seeded at 106 cells/mm^2^ density on 60 mm plastic dishes. After 16 h of simulated reperfusion, dead and live cells were collected according to the same protocol used in Section 2.8. Next, to permeabilize cell membranes, cold methanol was added to the live and dead cell mixture for 24 h, at −20 °C. RNAse (0.1 mg/mL) was then added to the samples for 1 h at room temperature. Finally, propidium iodide (1 mg/mL) was added to cells and apoptosis was determined by flow cytometry using a BD FACSCanto (Becton Dickinson & Company, Franklin Lakes, NJ, USA). Propidium iodide marks condensed chromatin and/or fragmented DNA in apoptotic bodies giving a low intensity signal (sub-G1 population), under the prominent G1 signal of living cells with integral DNA. A total of 5000 cells/sample were analyzed.

### 2.11. Western Blot Analysis

For protein content analysis, CF were seeded at a 106 cells/mm^2^ density on 60 mm plastic dishes. At the end of simulated reperfusion, cells were washed three times with cold PBS, followed by addition of RIPA lysis buffer with protease and phosphatase inhibitors. Samples were centrifuged at 252× *g* for 10 min at 4 °C, and supernatants were collected. Total protein concentration of samples was determined using the Bradford reagent, and absorbance was measured to 595 nm in an Epoch UV-Vis Spectrophotometer (BioTek Instruments, Inc., Winooski, VT, USA). We used 25 μg of total protein sample, which was separated by sodium dodecyl sulfate polyacrylamide gel electrophoresis (SDS-PAGE) using 10–20% acrylamide/bis-acrylamide gels run for 1.5–2 h. at constant 70–100 V. Proteins were then electro-transferred to a nitrocellulose membrane for 1–1.5 h with a 0.35 A constant current. Membrane was blocked with non-fat milk (5% *w/v*) for 1 h. Primary antibodies against p-ERK1/2, ERK1/2, p-Akt, Akt, p-p38, p38, p-JNK, JNK, pro-caspase 3, pro-caspase 9, Bax, Bcl-xl, α-SMA, COL1A1 (dilution 1:1000), or GAPDH (dilution 1:2000) were incubated overnight at 4 °C. Secondary antibodies for anti-rabbit IgG or anti-mouse IgG conjugated with HRP (dilution 1:5000) were incubated for 1.5 h at room temperature. Membrane was exposed to the ECL reagent and revealed in the C-DiGit Chemiluminescent Western Blot Scanner (LI-COR Biosciences, Lincoln, NE, USA). Images and blots were analyzed and quantified using the Image Studio™ software (LI-COR Biosciences, Lincoln, NE, USA).

### 2.12. Evaluation of Cell Migration by Wound Healing Assay

CF were seeded 156 cells/mm^2^ density on 35 mm plastic dishes in DMEM-F12 containing FBS (10%) and penicillin-streptomycin-amphotericin B and incubated during 24 h to allow proliferation until confluence was reached. Subsequently, cells were washed two times with PBS and incubated with DMEM-F12 for 24 h. Next, simulated ischemia was performed for 6 h, followed by a scratch made using a 200 µL tip in fibroblast monolayers at the beginning of simulated reperfusion. BrdU (100 μM) was used as a proliferation inhibitor, allowing CF to migrate without proliferating in the presence of FBS (10%). After 24 h, cells were stained with crystal violet (0.3% *w/v*) for 20 min at room temperature. After washing and drying plates, images were obtained from four fields per plate using an optic microscope. Scratched areas per image were analyzed with ImageJ software (LI-COR Biosciences, Lincoln, NE, USA). and the mean of four fields of scratched areas per plate was used for data analysis. All values of mean scratched area were normalized with respect to the value of mean scratched area of control groups with cells incubated in DMEM-F12.

### 2.13. Statistical Analysis

All data are presented as mean ± standard error of the mean (S.E.M.), of at least three independent experiments, and were analyzed using GraphPad Prism (GraphPad, San Diego, CA, USA) version 5.01 software. The differences between two experimental groups were evaluated by paired Student’s t-test. The differences between three or more experimental groups were evaluated by a one-way analysis of variance (ANOVA) followed by a Tukey post-test. The differences between two experimental groups at each time were evaluated by two-way ANOVA, followed by a Bonferroni post-test. Statistical significance was accepted at *p* < 0.05.

## 3. Results

### 3.1. Individual Effects of Ascorbic Acid, Deferoxamine, and N-Acetylcysteine on Viability of Cardiac Fibroblasts after Simulated Ischemia/Reperfusion

**Figure 1:**
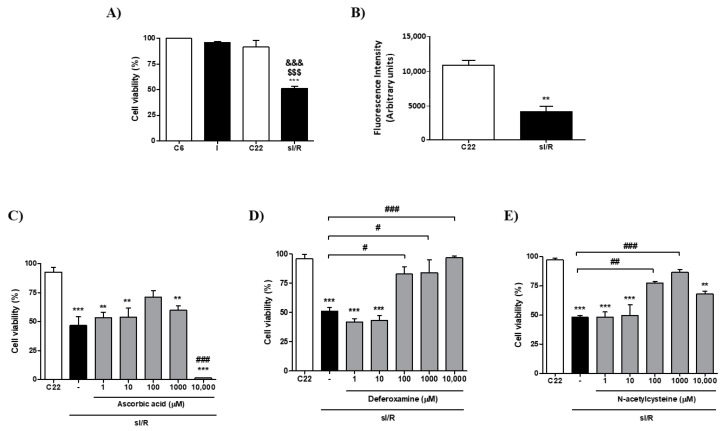
Effects of ascorbic acid, deferoxamine and N-acetylcysteine, at different concentrations, on viability of cardiac fibroblasts exposed to simulated ischemia/reperfusion. Cardiac fibroblasts were exposed to 6 h simulated ischemia, followed by 16 h simulated reperfusion (sI/R). (**A**) Cell viability was quantified as a percentage (%) of number of cells after 6 h normoxia (C6, 100%) by cell count after trypan blue staining (*n* = 3). (**B**) Cell viability was quantified as fluorescence intensity using the resazurin reduction assay (*n* = 3). At the beginning of simulated reperfusion, ascorbic acid (**C**), deferoxamine (**D**) and N-acetylcysteine (**E**) were added using 10,000; 1000; 100; 10; and 1 µM. Cell viability was quantified as the percentage (%) of number of cells after 6 h normoxia (100%) by cell count after trypan blue staining (*n* = 3). The results are expressed as mean ± S.E.M. &&& *p* < 0.001 vs. C6; $$$ *p* < 0.001 vs. I (cells after 6 h simulated ischemia); *** *p* < 0.001 and ** *p* < 0.01 vs. C22 (control cells after 22 h normoxia); ### *p* < 0.001, ## *p* < 0.01, and # *p* < 0.05 vs. sI/R.

In order to study the cytoprotective effects of antioxidants, we first validated our model by subjecting neonatal rat CF to 6 h of simulated ischemia, followed by 16 h of simulated reperfusion and we measured cell viability using trypan blue. The results show that after sI/R, cell viability was significantly reduced compared to normoxic controls (*p* < 0.001; Figure 1A), which was corroborated using the resazurin reduction assay (*p* < 0.01; Figure 1B). We then tested administration ascorbic acid (A), deferoxamine (D), and N-acetylcysteine (N) at 10,000; 1000; 100; 10; and 1 µM at the beginning of reperfusion and evaluated cell viability using trypan blue. None of the three antioxidants had any effect on CF viability at 10 and 1 µM after sI/R. (Figure 1C–E). However, treatment with D increased cell viability at 10,000; 1000; and 100 µM, while addition of N increased cell viability at 1000 and 100 µM, but not at 10,000 µM (Figure 1D,E, respectively). Administration of A increased cell viability only at 100 µM (non-significant), whereas 10,000 µM further reduced CF viability after sI/R, compared to untreated cells (*p* < 0.001; Figure 1C).

### 3.2. Ascorbic Acid, Deferoxamine, and N-Acetylcysteine Association Increased Cell Viability and Reduced Intracellular ROS Production in Cardiac Fibroblasts Subjected to Simulated Ischemia/Reperfusion

**Figure 2:**
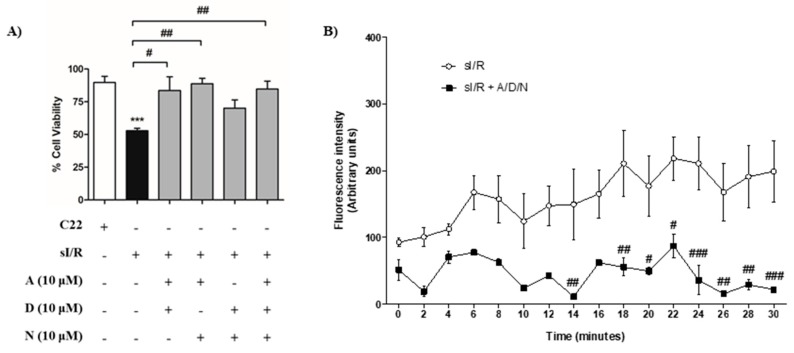
The association of ascorbic acid, deferoxamine, and N-acetylcysteine, at 10 µM each, increased cell viability and decreased intracellular reactive oxygen species (ROS) generation in cardiac fibroblasts exposed to simulated ischemia/reperfusion. (**A**) Cardiac fibroblasts were exposed to 6 h simulated ischemia followed by 16 h simulated reperfusion (sI/R). Associations between ascorbic acid (A), deferoxamine (D), and N-acetylcysteine (N), using 10 μM of each antioxidant, were added at the beginning of simulated reperfusion. Cell viability was quantified as a percentage (%) of number of cells after 6 h normoxia (100%) by cell count after trypan blue staining (*n* = 5). (**B**) Cardiac fibroblasts were exposed to 6 h simulated ischemia followed by 30 min simulated reperfusion (sI/R). At the end of ischemia, cells were incubated with 2′,7′-dichlorofluorescin diacetate and then treated with A/D/N using 10 μM of each antioxidant at the onset of simulated reperfusion. Intracellular ROS generation was measured as the fluorescence intensity in a time course during the first 30 min of simulated reperfusion (*n* = 3). The results are expressed as mean ± S.E.M. *** *p* < 0.001 vs. C22 (control cells after 22 h normoxia); ### *p* < 0.001, ## *p* < 0.01 and # *p* < 0.05 vs. sI/R. Symbol “+” represents presence of condition and symbol “-” represents absence of condition.

Next, we sought to evaluate whether the pharmacological association of A, D, and N could provide synergistic protection by using a low concentration of each antioxidant that had no effect on cell viability when administered separately. To achieve this, CF were exposed to sI/R and we simultaneously administered combinations of the three antioxidants at the onset of reperfusion at 1 and 10 μM, and then measured cell viability using trypan blue. Our results show that treatments with the associations of A/D, A/N, and A/D/N, but not D/N, increased cell viability after sI/R at 10 μM each, when compared to untreated conditions (Figure 2A). However, only the A/D association protected at 1 μM (Supplementary Figure S1). Based on these findings, we decided to study the cytoprotective effect of the A/D/N association at 10 μM given its potential to provide more robust protection by reducing oxidative stress by three different mechanisms, unlike double associations of these antioxidants. In addition, we observed that the A/D/N association at 10 μM each significantly reduced intracellular ROS production after sI/R, compared to untreated cells, as measured with a DCFH-DA probe (Figure 2B). Therefore, we used the joint administration of 10 μM of A/D/N for the rest of the experiments.

### 3.3. Association of Ascorbic Acid, Deferoxamine, and N-Acetylcysteine Reduced Apoptosis of CF Exposed to Simulated Ischemia/Reperfusion

**Figure 3:**
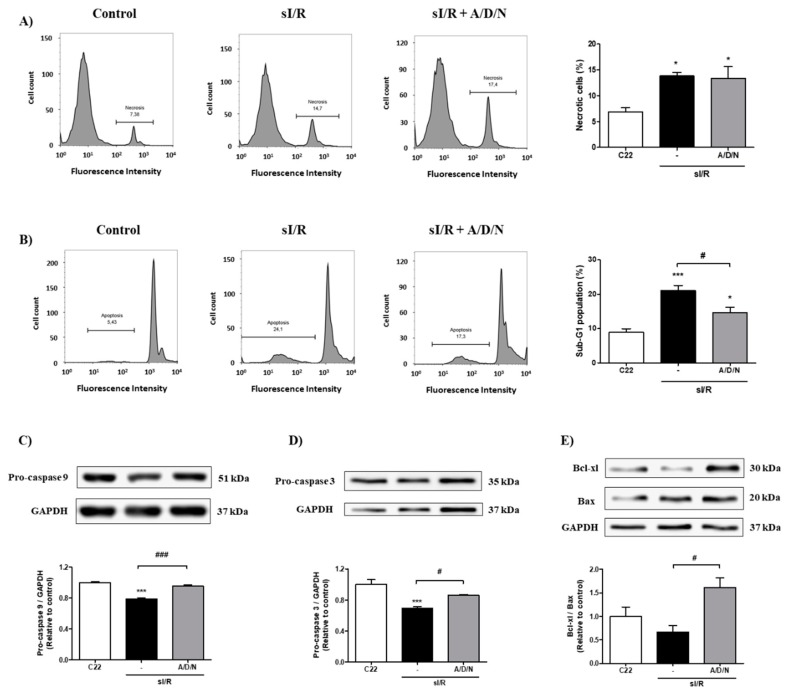
The association of ascorbic acid, deferoxamine and N-acetylcysteine reduced apoptosis induced by simulated ischemia/reperfusion in CF. Cells were exposed to 6 h simulated ischemia, followed by 16 h simulated reperfusion (sI/R). Cells were treated with the association of ascorbic acid, deferoxamine, and n-acetylcysteine (A/D/N) using 10 μM of each antioxidant at the onset of simulated reperfusion. (**A**) The percentage (%) of necrotic cells was quantified by flow cytometry using propidium iodide (right panel), with representative histograms of each experimental group (left panel; *n* = 4). (**B**) The percentage (%) of the sub-G1 population was quantified by flow cytometry using propidium iodide (right panel), with representative histograms of each experimental group (left panel; *n* = 5). (**C**–**E**) show representative Western blots (upper panel) and densitometric analysis (lower panel) of pro-caspase 9 (*n* = 4), pro-caspase-3 (*n* = 5), and Bcl-xl/Bax ratio (*n* = 3), respectively. Glyceraldehyde 3-phosphate dehydrogenase (GAPDH) was used as a loading control. The results are expressed as mean ± S.E.M. *** *p* < 0.001 and * *p* < 0.05 vs. C22 (control cells after 22 h normoxia); ### *p* < 0.001 and # *p* < 0.05 vs. sI/R.

To confirm our findings of cell death types, we measured necrosis and apoptosis after sI/R using flow cytometry analysis with propidium iodide staining. The results indicate that antioxidant association had no effect on necrosis induced by sI/R (Figure 3A). However, treatment with A/D/N induced a decrease in the sub-G1 population of CF exposed to sI/R, compared to untreated conditions (*p* < 0.05; Figure 3B). To further corroborate that the A/D/N association can inhibit apoptosis induced by sI/R in CF, we determined the protein levels of pro-caspase 9 and pro-caspase 3, as well as the Bcl-xl/Bax ratio after treatment with A/D/N. Our data shows that cells exposed to sI/R presented lower levels of pro-caspases 9 and 3, as compared with normoxic cells (both *p* < 0.001). This result suggests an induction of apoptosis (Figure 3C,D), but administration of the A/D/N association prevented this effect and increased the Bcl-xl/Bax ratio (*p* < 0.05), compared to untreated CF after sI/R (Figure 3C–E). In addition, to test whether these findings were reproduced in a different cell type, we subjected primary neonatal rat cardiomyocytes to sI/R and then treated them with A/D/N. Our results show that A/D/N increased viability of cardiomyocytes (Supplementary Figure S2A) and increased the levels of pro-caspases 9 and 3 (Supplementary Figure S2B,C).

### 3.4. Association of Ascorbic Acid, Deferoxamine and N-Acetylcysteine Activated the Pro-Survival Kinases ERK1/2 and Akt and Reduced the Phosphorylation of the Pro-Apoptotic Proteins p38.MAPK and JNK Induced by Simulated Ischemia/Reperfusion in CFs

**Figure 4:**
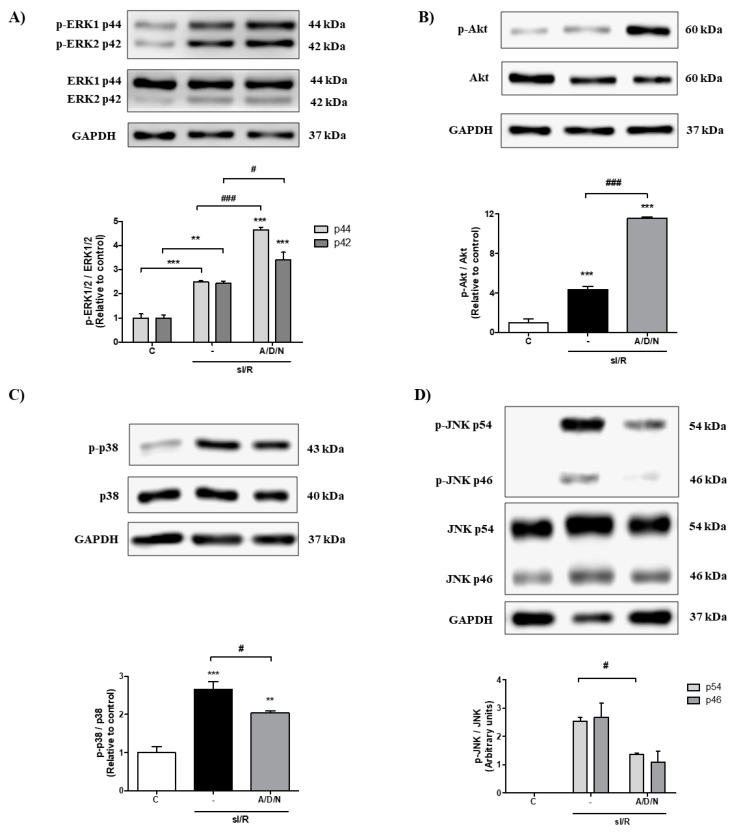
The association of ascorbic acid, deferoxamine, and N-acetylcysteine increased the activation of the pro-survival kinases ERK1/2 and Akt, and reduced activation of the pro-apoptotic proteins p38 and JNK in cardiac fibroblasts exposed to simulated ischemia/reperfusion. Cardiac fibroblasts were exposed to 6 h simulated ischemia, followed by 10 min of simulated reperfusion (sI/R). Cells were treated with the association of ascorbic acid, deferoxamine and N-acetylcysteine (A/D/N) using 10 μM of each antioxidant at the onset of simulated reperfusion. (**A**–**D**) show representative Western blots (upper panel) and densitometric analysis (lower panel) of p-ERK1/2 and ERK1/2 (*n* = 3), p-Akt and Akt (*n* = 3), p-p38 and p38 (*n* = 4), and p-JNK and JNK (*n* = 3), respectively. GAPDH was used as a loading control. The results are expressed as mean ± S.E.M. *** *p* < 0.001 and ** *p* < 0.01 vs. C (control cells after 70 min normoxia); ### *p* < 0.001 and # *p* < 0.05 vs. sI/R.

In order to pursue the mechanism by which the association of antioxidants conferred its protective effect, we sought to evaluate the signaling pathways associated with cell survival. To test this, we determined by Western blot the early activation of ERK1/2 and Akt in response to administration of A/D/N in CF exposed to sI/R. The results show that sI/R significantly increased phosphorylation of ERK1/2 and Akt after 10 min of simulated reperfusion, compared to normoxic conditions, but this effect was further potentiated by the treatment with the antioxidant association (Figure 4A,B). In addition, sI/R also elicited early phosphorylation of the pro-apoptotic proteins p38 and JNK in comparison with normoxic conditions, but this effect was diminished by administration of A/D/N (*p* < 0.05; Figure 4C,D).

### 3.5. Association of Ascorbic Acid, Deferoxamine, and N-Acetylcysteine Prevented the Loss of Function Associated with Tissue Repair Induced by Simulated Ischemia/Reperfusion in CF

**Figure 5:**
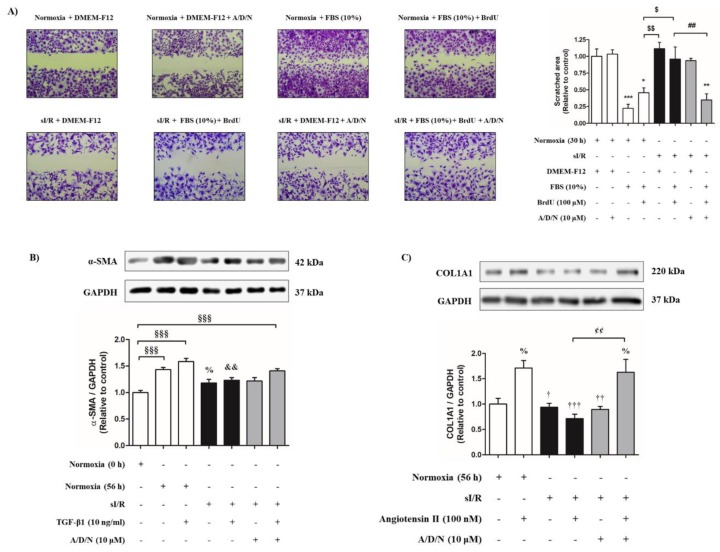
The association of ascorbic acid, deferoxamine, and N-acetylcysteine prevents the loss of serum-induced migration, TGF-β1-mediated differentiation and angiotensin II-induced pro-collagen I synthesis induced by simulated ischemia/reperfusion in CF. (**A**) Cells were exposed to 6 h simulated ischemia, followed by 24 h simulated reperfusion (sI/R). Cells were treated with the association of ascorbic acid, deferoxamine, and n-acetylcysteine (A/D/N) using 10 μM of each antioxidant at the onset of simulated reperfusion. A scratch was made on a cell monolayer prior to simulated reperfusion with DMEM-F12, FBS (10%), or FBS (10%) + BrdU (100 μM). Representative images of each experimental group (left panel) and quantification of area (right panel) are shown (*n* = 3). (**B**) Cardiac fibroblasts were exposed to 6 h simulated ischemia, followed by 48 h simulated reperfusion (sI/R). At the onset of simulated reperfusion, cells were treated with the association of A/D/N using 10 μM of each antioxidant and stimulated with or without TGF-β1 (10 ng/mL). Representative Western blots (upper panel) and densitometric analysis (lower panel) of α-SMA and GAPDH as loading control are shown (*n* = 5). (**C**) Cardiac fibroblasts were exposed to 6 h simulated ischemia, followed by 48 h simulated reperfusion (sI/R). At the beginning of simulated reperfusion, cells were treated with the association of A/D/N using 10 μM of each antioxidant and stimulated with or without angiotensin II (100 nM). Ascorbic acid (100 nM) was added to all experimental groups as a co-factor in pro-collagen type I synthesis. Representative Western blots (upper panel) and densitometric analysis (lower panel) of COL1A1 and GAPDH as loading control are shown. The results are expressed as mean ± S.E.M. *** *p* < 0.001, ** *p* < 0.01 and * *p* < 0.05 vs. Normoxia (30 h) + DMEM-F12; $$ *p* < 0.01 and $ *p* < 0.05 vs. Normoxia (30 h) + FBS (10%) + BrdU; ## *p* < 0.01 vs. sI/R + FBS (10%) + BrdU; §§§ *p* < 0.001 vs. Normoxia (0 h); % *p* < 0.05 vs. Normoxia (56 h); && *p* < 0.01 vs. Normoxia (56 h) + TGF-β1; ††† *p* < 0.001, †† *p* < 0.01 and † *p* < 0.05 vs. Normoxia (56 h) + angiotensin II; ¢¢ *p* < 0.01 vs. sI/R + angiotensin II. COL1A1 = alpha-1 type 1 collagen; TGF-β1 = transforming growth factor beta 1; α-SMA = alpha smooth muscle actin; FBS = fetal bovine serum; BrdU = 5-bromo-2′-deoxyuridine. Symbol “+” represents presence of condition and symbol “-” represents absence of condition.

Finally, we studied whether the A/D/N association can protect CF function associated with detriments in migration, differentiation and collagen secretion, after sI/R. To assess cell migration, we performed the wound healing assay using FBS (10%) to induce migration of CF to the scratched area during simulated reperfusion, and we applied BrdU to inhibit the proliferation induced by FBS. Figure 5A shows that, in normoxic conditions, CF migrated in the presence of FBS (10%), with or without BrdU, reducing the scratched area over 50%, compared with control cells (*p* < 0.001). However, cells did not migrate in the presence of the A/D/N association. Then, we tested whether CF exposed to sI/R can migrate in the presence of FBS (10%) + BrdU. Figure 5A shows that, after 6 h simulated ischemia followed by 24 h simulated reperfusion, CF did not migrate in the presence of FBS (10%) + BrdU. In addition, the A/D/N association by itself did not modify the migration of CF exposed to sI/R. However, when the cells were exposed to sI/R and treated with A/D/N in the presence of FBS (10%) + BrdU, migration was significantly increased compared to normoxic control cells without FBS (10%; *p* < 0.01), and compared to cells under sI/R and in presence of FBS (10%) + BrdU (*p* < 0.01; Figure 5A).

In addition, we induced CF-to-CMF differentiation by incubating CF in the presence or absence of TGF-β1 (10 ng/mL) during simulated reperfusion, and measured α-SMA (alpha smooth muscle actin)—a differentiation protein level marker—by Western blot after 48 h. Figure 5B shows that α-SMA protein content is increased after 6 h simulated ischemia, followed by 48 h simulated reperfusion with or without TGF-β1 administration, compared to control conditions (*p* < 0.001), but these effects were inhibited after sI/R. Addition of A/D/N alone did not increase α-SMA protein levels in CF after sI/R, but co-administration of antioxidants with TGF-β1 restored the cytokine’s differentiating effect, compared to control conditions (*p* < 0.001; Figure 5B).

Furthermore, pro-collagen I synthesis was assessed by stimulation of CF with angiotensin II (100 nM) during simulated reperfusion for 48 h. Western blot analysis revealed increased angiotensin II induced-pro-collagen I protein levels with respect to control conditions (*p* < 0.05), which were significantly inhibited after 6 h simulated ischemia followed by 48 h simulated reperfusion (*p* < 0.001; Figure 5C). A/D/N alone also had no effect in pro-collagen I protein levels in CF after sI/R, but joint administration with angiotensin II restored the production of pro-collagen I induced by this peptide, in comparison to normoxic conditions (*p* < 0.05), and to cells under sI/R treated with angiotensin II (*p* < 0.01; Figure 5C).

## 4. Discussion

The main findings of the present study showed that the pharmacological association of A, D, and N: (a) increased cell viability in CF exposed to sI/R using a lower concentration than those of each antioxidant which, separately, did not show cell viability protection, (b) reduced intracellular ROS production and decreased apoptotic cell death induced by sI/R, (c) activated the pro-survival kinases ERK1/2 and Akt, but inhibited the pro-apoptotic p38 and JNK kinases, and (d) recovered CF function associated with wound repair induced by sI/R by restoring serum-induced migration, TGF-β1-mediated differentiation of CF into CMF and angiotensin II-induced pro-collagen I synthesis.

The deleterious effects of reperfusion injury and oxidative stress on the functional capacity of CF could negatively affect the optimal development of myocardial repairing after tissue damage [26]; therefore, cytoprotection of fibroblasts is of the utmost importance. Currently, there are multiple strategies to protect the myocardium from I/R injury, but the translation of these therapies from bench to bedside has proved challenging. Combinations of therapies with synergistic effects, as well as protection of all cardiac cell types and not just cardiomyocytes, are believed to be essential to achieve cardioprotection in the clinical arena [27,28].

Our preliminary studies revealed that A, D, and N separately increase the viability of CF exposed to sI/R in a concentration-dependent manner, at high concentrations (≥100 µM). However, 10 mM of A was cytotoxic due to its pro-oxidant activity at higher concentrations, which has been demonstrated in murine tumors [29]. Moreover, these antioxidants, separately, did not protect against sI/R injury at 1 and 10 µM, but when they were associated (dual or triple combinations) at 10 µM each, showed a synergistic cytoprotective effect. Previous studies have thoroughly established that A, D, and N -alone or in dual combination- yield cardioprotective effects [17,18,19,20,21,22,30,31,32,33]. Nonetheless, this is the first study that shows protection elicited by A/D/N administration in CF after sI/R. In addition, in vivo assays of MI will also be necessary to clarify whether the A/D/N association could prevent cardiac cell death, in order to reduce infarct size and finally, improve wound healing. Conversely, a study reported by Nikas et al., [34] showed that combined intravenous administration of A, D, and N, 15 min before and 5 min after reperfusion, with the same dose, was unable to reduce infarct size, the deleterious effects on ventricular function parameters, or oxidative stress markers in an in vivo model of myocardial reperfusion injury in pigs. Although the authors use a reliable I/R model tested in large animals, they only tested a single dose of each antioxidant (3–3.5 g A, 1.8–2.1 g D, and 3–3.5 g N) and did not measure the plasma levels reached by A, N, and D to compare with the results of the present study.

We speculated that the synergistic cytoprotective effects of the associations between A/D, A/N, and A/D/N, but not D/N (using each antioxidant at 10 µM) on the viability of CF exposed to sI/R, could be intrinsically related to a favorable pharmacological interaction between A, N and D, in the context of myocardial reperfusion injury elicited by oxidative stress [6,8]. Intracellular GSH -an endogenous antioxidant molecule- can reduce oxidized dehydroascorbate to ascorbate (an ionized form of A), which increases the bioavailability of ascorbate to interact against ROS [35]. Moreover, the decrease in intracellular GSH levels may be replenished by N, and D can chelate an excess of catalytic free iron (which increases during ischemia and cardiac reperfusion from cell lysis), thereby decreasing the production of hydroxyl radicals through the Fenton reaction, and preventing the pro-oxidant interaction of iron with A [36]. Thus, the A/D/N association has the advantage of improving the antioxidant response against the increase of intracellular ROS during simulated reperfusion, by providing three different mechanisms of antioxidant action. Finally, the use of lower concentrations of each antioxidant, compared to those used in independent administration to achieve a pharmacological effect in CF, ensured us that possible toxic effects could be low or absent.

Moreover, the A/D/N combination also activated ERK1/2 and Akt, which are key components of the reperfusion injury salvage kinases (RISK) pathway and therefore, essential to protect cardiac cells exposed to sI/R from reperfusion-induced cell death [37,38]. Currently, there are a few studies linking the activation of the RISK pathway to A and N [18,31,39], and therefore, our findings further support a nexus between this well-known survival signaling pathway and the cytoprotective effect of antioxidant association. However, additional studies are necessary to establish cause-effect mechanisms in order to evaluate whether inhibition of the RISK pathway results in the loss of protection conferred by the A/D/N triad. Additionally, treatment with A/D/N triggered anti-apoptotic effects in CF exposed to sI/R by reducing the phosphorylation of p38 and JNK, which are important players in apoptosis in the context of cardiac I/R [7,40]. This signaling pathway may be triggered by the apoptosis signal-regulating kinase 1 (ASK1), which can be activated by ROS, and consequently, induce apoptosis [41]. Further research is also required to thoroughly assess the molecular mechanisms by which the A/D/N antioxidant association activates the RISK pathway and also inactivates p38 and JNK.

The A/D/N association reduced the activation of caspases 9, 3 and increased the Bcl-xl/Bax ratio. Moreover, using the same sI/R protocol, protection was also observed in cardiomyocytes treated with A/D/N. These effects were further confirmed by the assessment of pro-apoptotic proteins, suggesting that our pharmacological approach may protect the whole myocardium and not only fibroblasts. These results are consistent with the previously reported anti-apoptotic effects conferred by A, D, and N [18,19,31,42], and contribute to a better characterization of the role of oxidative stress as a pharmacological target in apoptosis induced by I/R. Furthermore, we did not observe a protective effect of A/D/N against necrosis, which could initially suggest that there are factors other than oxidative stress (e.g., decreased energy metabolism) that might contribute most importantly to this type of cell death during simulated reperfusion. However, the RISK pathway protects against cell death by apoptosis and necrosis, and in our in vitro model this survival pathway is activated by the A/D/N association in cardiac fibroblasts; therefore, future studies will be required to understand this differential protection against these two types of cell death. Moreover, given the normal limitations of the cell death assays we used, future research should thoroughly assess the exact type of cell death prevented by the A/D/N association.

CF migration is a key step in the wound repair process, allowing CF located in close areas to the infarction zone to migrate and repopulate the necrotic area. Chemokines (Fractalkine/CX3CL1), growth factors (TGF-β and fibroblast growth factor), and cytokines (interleukin-1β, tumor necrosis factor α and cardiotrophin-1) secreted from other cells types further secrete ECM, cytokines and chemokines (as MCP-1) to induce immune cell migration and ensure fast tissue repair [9,43]. Due to its high content of embryonic growth-promoting factors, FBS is used to induce in vitro migration in CF and other cell types [44,45]. In our study, cellular injury caused by sI/R triggered the impairment of the FBS-induced migratory capacity of CF. Similar results were found in adult rat CF, where stimulation with hydrogen peroxide 10 and 100 μM showed that migration induced by a fibronectin gradient was delayed, compared to untreated control cells [46]; these results highlight the deleterious effect of oxidative stress on migration of these cells and suggest the protective molecular mechanism of the A/D/N association.

One of the main characteristics of CF is their ability to differentiate into CMF, which are characterized by a pro-fibrotic phenotype [47]. These cellular changes are induced by several stimuli, such as TGF-β1, interleukin-10, thrombospondin-1, angiotensin II, stimulation of injury-site cardiomyocytes, and vascular cells, among others [26]. CMF are the main secretory source of ECM proteins, as well as matrix metalloproteases, in cardiac fibrotic remodeling [26]. In our study, we induced spontaneous CF-to-CMF differentiation, which is due probably to the autocrine effects of TGF-β1 secreted by CF in culture, as well as treatment with TGF-β1. Both methods induced an increase in α-SMA levels in CF after 48 h of simulated reperfusion. Although TGF-β1 stimulation was not significantly greater than spontaneous differentiation, we and others have shown that TGF-β1 increases α-SMA levels in CF in a time-dependent manner [45,48]. Interestingly, this inhibitory effect of sI/R on the increase of α-SMA levels induced by TGF-β1 or spontaneous differentiation in CF has not been previously described. A similar effect has been observed in H9c2 cardiomyocytes and in corneal keratocytes, where hypoxia prevented the transformation to myofibroblasts induced by, an effect which was associated with changes in TGF-β1 signaling pathways [49,50]. Further studies are necessary to elucidate the effects of the A/D/N association on signaling pathways implicated in the effects of differentiation of CF to MCF induced by TGF-β1 after sI/R.

CF and CMF secrete and degrade various types of collagen to maintain ECM homeostasis. Among these, type I collagen is widely expressed in cardiac tissue of mammals and forms thick and stiff fibers [26]. Angiotensin II is a peptide known to elicit cardiac fibrosis [51,52] by stimulating CF collagen production and secretion [53,54]. In our CF in vitro model, sI/R prevented the increase of pro-collagen I synthesis triggered by angiotensin II and did not induce pro-collagen I production by itself. These results were corroborated by Siwik et al., [55] who demonstrated that oxidative stress decreases fibrillar collagen synthesis in CF. Interestingly, previous reports have shown that 72 h of hypoxia induce an increase in pro-collagen type I α mRNA and protein levels in human CF [56], while 6 h of hypoxia also increased collagen I levels in adult rat CF [57]. Additionally, another study found that 1 h of hypoxia followed by 12 h reoxygenation increases the secretion of soluble collagen from neonatal rat CF [58]. These differences can be attributed to various factors, such as duration of hypoxia/reoxygenation, age, and species from which these cells originate.

Our observations that the A/D/N association prevents cell death, reduces oxidative stress and recovers cellular functions associated with tissue repair induced by sI/R in CF certainly supports the previously described cardioprotective effects of A, D, and N; either separately or combined, on ventricular function in animal models of myocardial I/R [17,18,19,30,32,33].

## 5. Conclusions

Overall, our findings indicate, for the first time, that the association of A, D, and N protects CF from cell death and recovers pro-wound healing function damaged by sI/R. We used a low concentration of each antioxidant, which did not increase cell viability when administered separately. Moreover, this effect may be mediated by activation of the RISK pathway and inhibition of the pro-apoptotic proteins p38 and JNK, suggesting that pharmacological association of these antioxidants may be a novel therapeutic strategy to protect the myocardium from reperfusion injury elicited by oxidative stress.

## Supplementary Materials

The following are available online at https://www.mdpi.com/2076-3921/8/12/614/s1. Figure S1: Effects of the association between ascorbic acid, deferoxamine and N-acetylcysteine, at 1 µM each, on viability of cardiac fibroblasts exposed to simulated ischemia/reperfusion. Cardiac fibroblasts were exposed to 6 h simulated ischemia, followed by 16 h simulated reperfusion (sI/R). Associations between ascorbic acid (A), deferoxamine (D) and N-acetylcysteine (N), using 1 μM of each antioxidant, were added at the beginning of simulated reperfusion. Cell viability was quantified as a percentage (%) of the number of cells after 6 h normoxia (100%) by cell count after trypan blue staining (*n* = 3). The results are expressed as mean ± S.E.M. *** *p* < 0.001 and * *p* < 0.05 vs. C22 (control cells after 22 h normoxia); ## *p* < 0.01 vs. sI/R. Figure S2: Effects of associations between ascorbic acid, deferoxamine and N-acetylcysteine, at 10 µM each, on viability and pro-caspases 9 and 3 protein levels of cardiomyocytes exposed to simulated ischemia/reperfusion. Cardiomyocytes were exposed to 6 h simulated ischemia, followed by 16 h simulated reperfusion (sI/R). Associations between ascorbic acid (A), deferoxamine (D) and N-acetylcysteine (N), using 10 μM of each antioxidant, were added at the beginning of simulated reperfusion. (**A**) Cell viability was quantified as a percentage (%) of the number of cells after 6 h normoxia (100%) by cell count after trypan blue staining (*n* = 3). (**B**) and (**C**) show representative Western blots (upper panel) and densitometric analysis (lower panel) of pro-caspase 9 (*n* = 4) and pro-caspase-3 (*n* = 4), respectively. GAPDH was used as a loading control. The results are expressed as mean ± S.E.M. *** *p* < 0.001 and ** *p* < 0.01 vs. C22 (control cells after 22 h normoxia). ### *p* < 0.001, ## *p* < 0.01 and # *p* < 0.05 vs. sI/R.

## Chemical compounds studied in this article

Angiotensin II (PubChem CID: 172198); Ascorbic acid (PubChem CID: 54670067); 5-Bromo-2′-deoxyuridine (PubChem CID: 6035); Deferoxamine mesylate (PubChem CID: 62881); 2,7-Dichlorodihydrofluorescein diacetate (2′,7′-dichlorofluorescin diacetate, PubChem CID: 77718); N-acetylcysteine (PubChem CID: 12035); Propidium iodide (PubChem CID: 104981); Resazurin (PubChem CID: 11077).

## Abbreviations

α-SMA = alpha smooth muscle actin; A/D/N = ascorbic acid, deferoxamine and N-acetylcysteine; A = ascorbic acid; BrdU = 5-bromo-2′-deoxyuridine; CF = cardiac fibroblast; CMF = cardiac myofibroblast; COL1A1 = alpha-1 type 1 collagen; DCFH-DA = 2′,7′-dichlorofluorescin diacetate; D = deferoxamine; ECM = extracellular matrix; ERK1/2 = extracellular-signal-regulated kinase1/2; FBS = fetal bovine serum; I/R = ischemia/reperfusion; JNK = c-Jun-N-terminal kinase; MI = myocardial infarction; N = N-acetylcysteine; RISK = reperfusion injury salvage kinases; ROS = reactive oxygen species; sI/R = simulated ischemia/reperfusion; TGF-β1 = transforming growth factor beta 1.
