# Supplementary material for: The Association of Ascorbic Acid, Deferoxamine and N-Acetylcysteine Improves Cardiac Fibroblast Viability and Cellular Function Associated with Tissue Repair Damaged by Simulated Ischemia/Reperfusion
Source: Antioxidants (Basel). 2019 Dec 3;8(12):614. doi: 10.3390/antiox8120614 (PMC6943610; doi:10.3390/antiox8120614)
Supplement: Supplementary file 1 [file antioxidants-08-00614-s001.pdf]

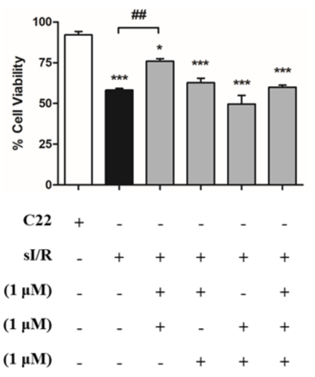

Figure S1. Effects of associations between ascorbic acid, deferoxamine, and N-acetylcysteine, at 1 μM each, on viability of cardiac fibroblasts exposed to simulated ischemia/reperfusion. Cardiac fibroblasts were exposed to 6 h simulated ischemia followed by 16 h simulated reperfusion (sI/R). Associations between ascorbic acid (A), deferoxamine (D), and N-acetylcysteine (N), using 1 μM of each antioxidant, were added at the beginning of simulated reperfusion. Cell viability was quantified as a percentage (%) of number of cells after 6 h normoxia (100%) by cell count after trypan blue staining ($n = 3$). The results are expressed as mean ± S.E.M. *** $p < 0.001$, * $p < 0.05$ vs. C22 (control cells after 22 h normoxia); ## $p < 0.01$ vs. sI/R.

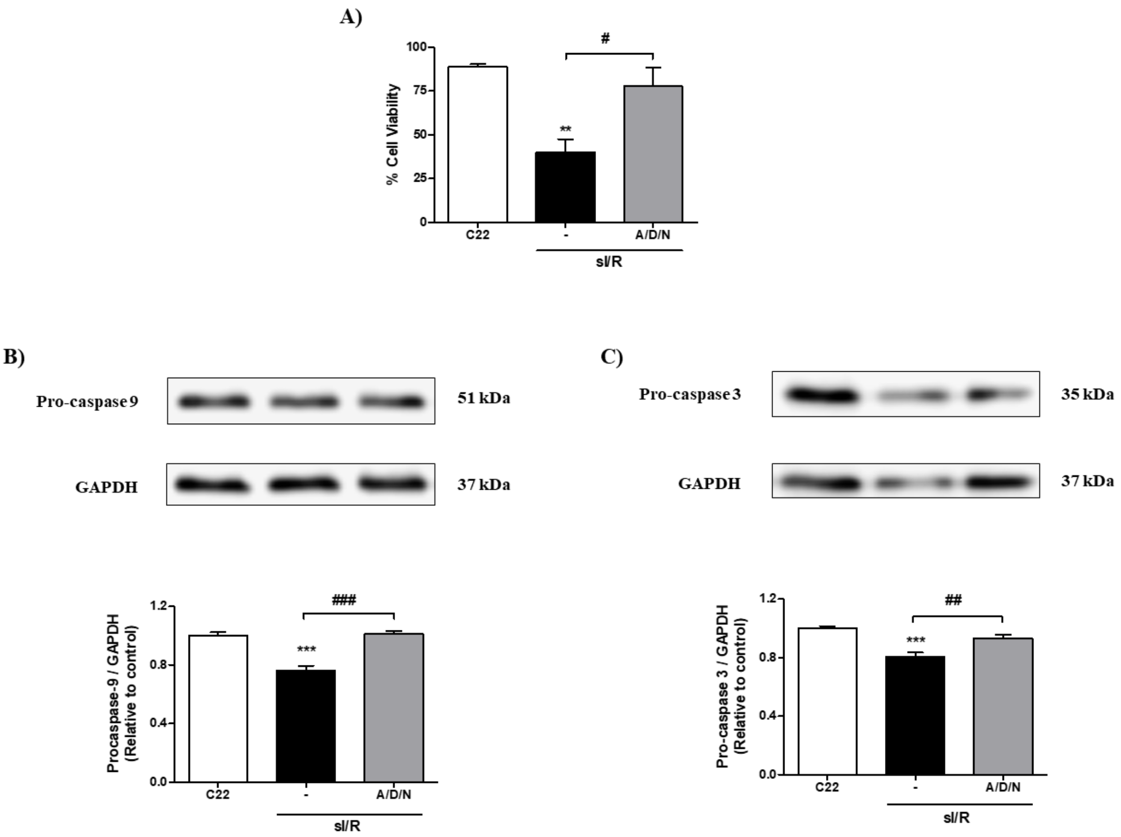

Figure S2. Effects of associations between ascorbic acid, deferoxamine, and N-acetylcysteine, at 10 μM each, on viability and protein levels of pro-caspases 9 and 3 of cardiomyocytes exposed to simulated ischemia/reperfusion. Cardiomyocytes were exposed to 6 h simulated ischemia followed by 16 h simulated reperfusion (sI/R). Associations between ascorbic acid (A), deferoxamine (D), and N-acetylcysteine (N), using 10 μM of each antioxidant, were added at the beginning of simulated reperfusion. **(A)** Cell viability was quantified as a percentage (%) of number of cells after 6 h normoxia (100%) by cell count after trypan blue staining (n = 3). **(B)** and **(C)** show representative Western blots (upper panel) and densitometric analysis (lower panel) of pro-caspase 9 (n = 4) and pro-caspase-3 (n = 4), respectively. GAPDH was used as loading control. The results are expressed as mean ± S.E.M. *** $p < 0.001$, ** $p < 0.01$ vs. C22 (Control cells after 22 h normoxia); ### $p < 0.001$, ## $p < 0.01$, # $p < 0.05$ vs. sI/R.
